# Supplementary figures and images for: Hypoxia-inducible factor 1 signaling drives placental aging and can provoke preterm labor
Source: eLife. 2023 Aug 23;12:RP85597. doi: 10.7554/eLife.85597 (PMC10446824; doi:10.7554/eLife.85597)

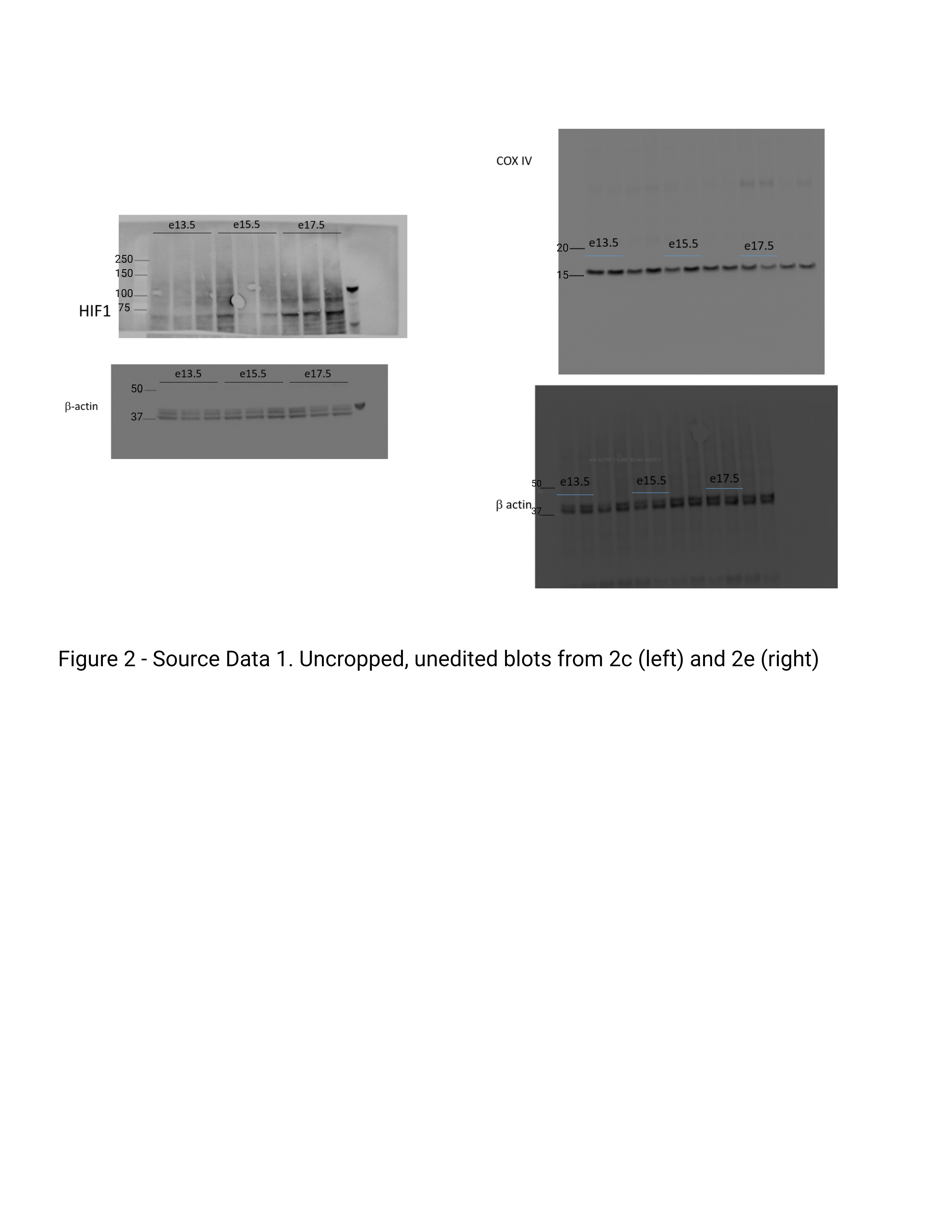

Supplement: Figure 2—source data 1. [file elife-85597-fig2-data1.zip › 85597Figure2-SourceData1.png]

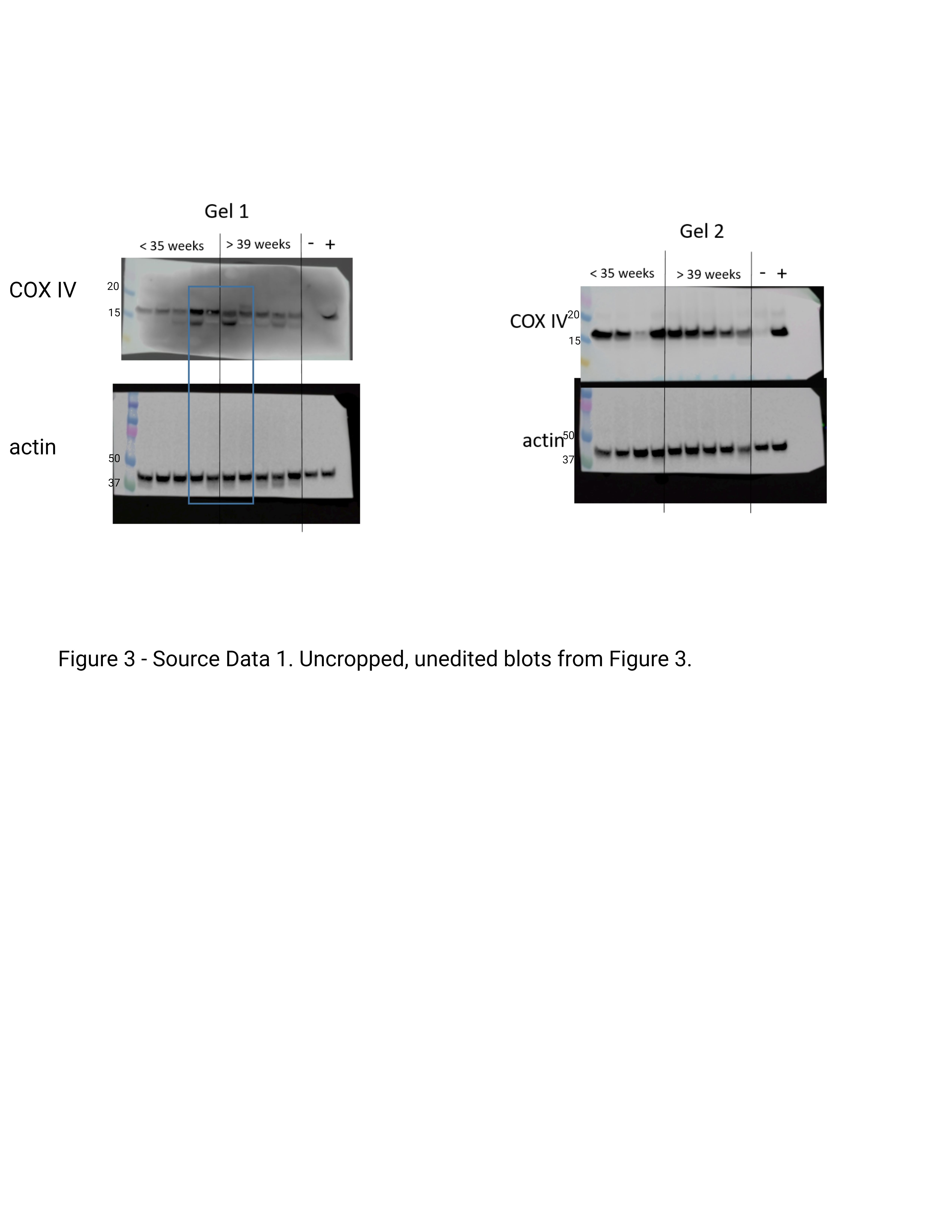

Supplement: Figure 3—source data 1. [file elife-85597-fig3-data1.zip › 85597Figure3-SourceData1.png]

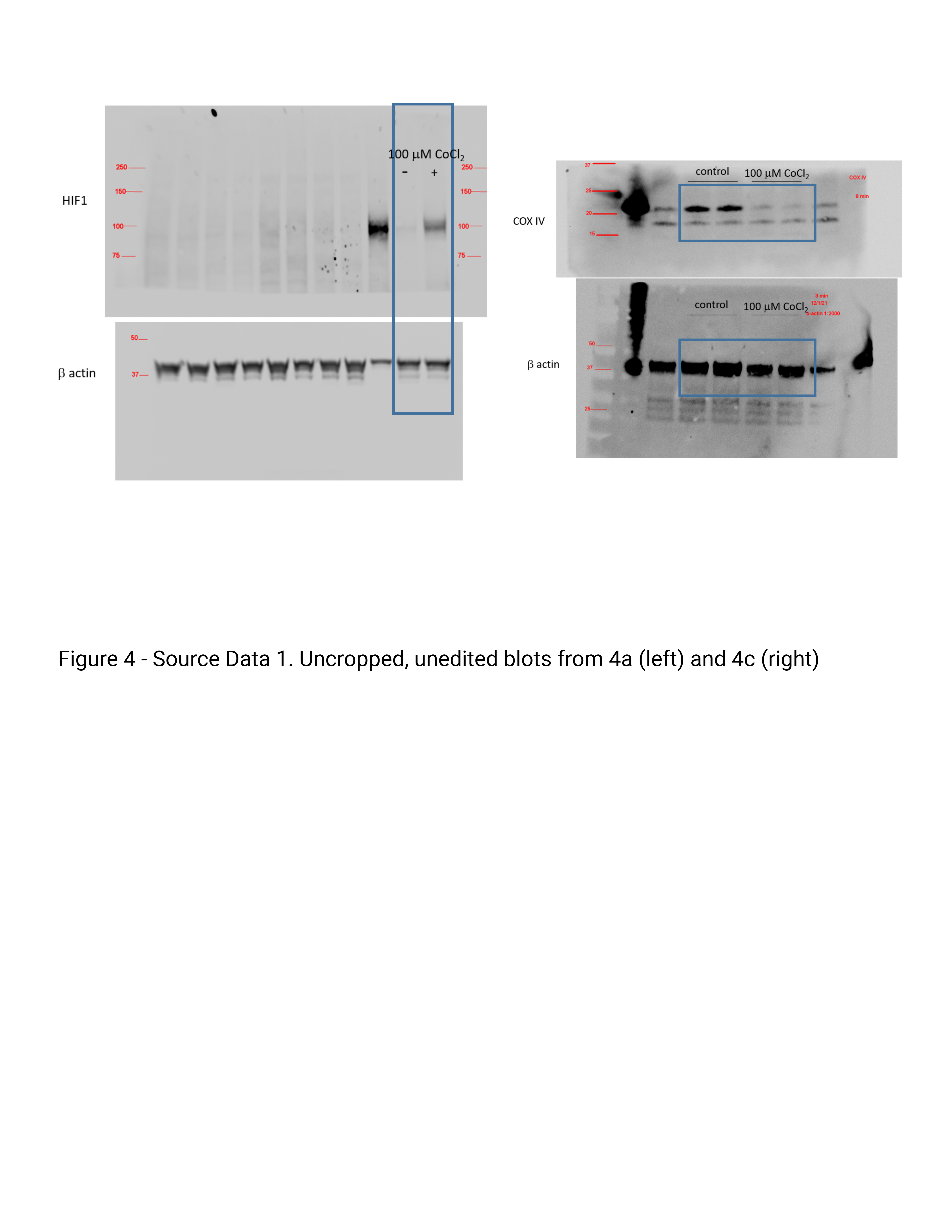

Supplement: Figure 4—source data 1. [file elife-85597-fig4-data1.zip › 85597Figure4-SourceData1.png]

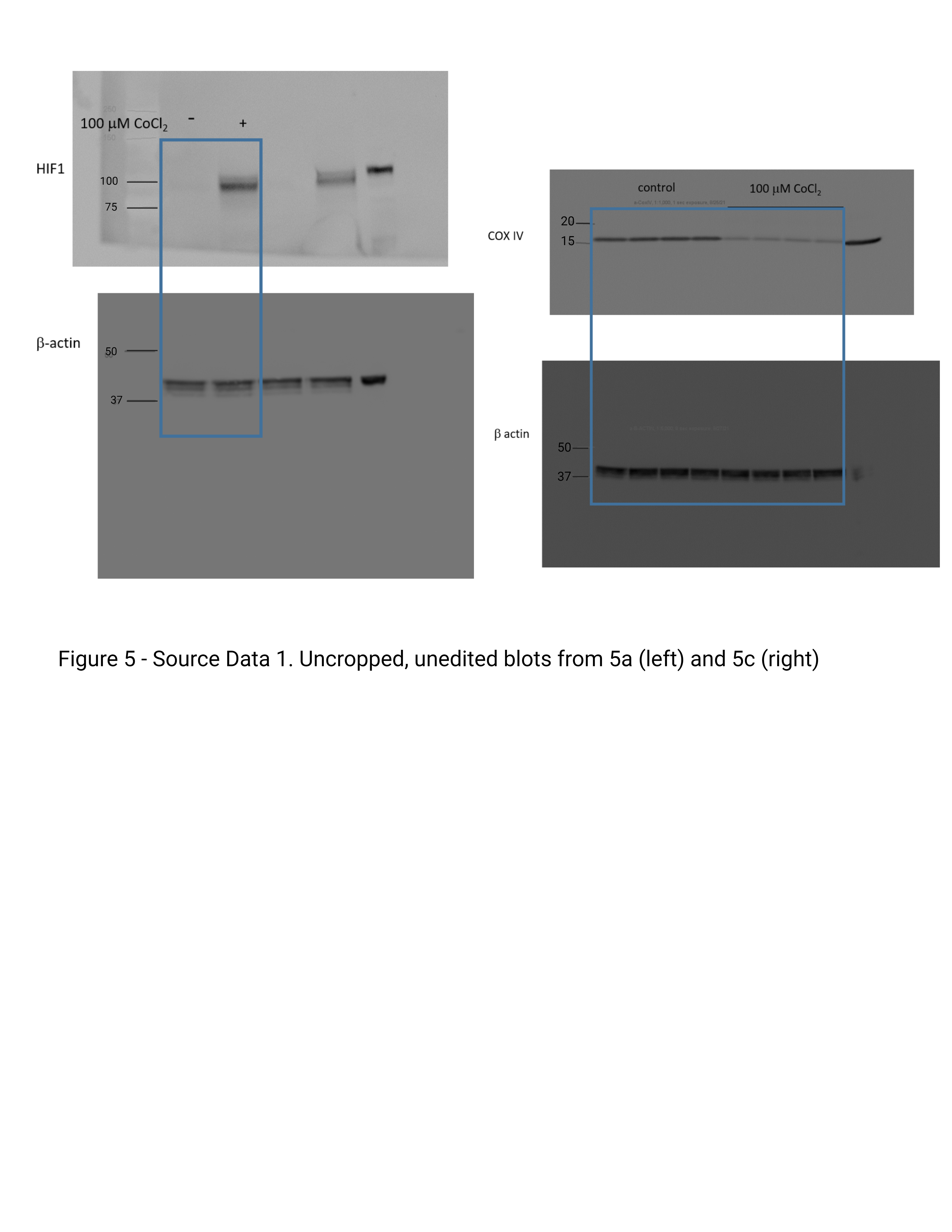

Supplement: Figure 5—source data 1. [file elife-85597-fig5-data1.zip › 85597Figure5-SourceData1.png]

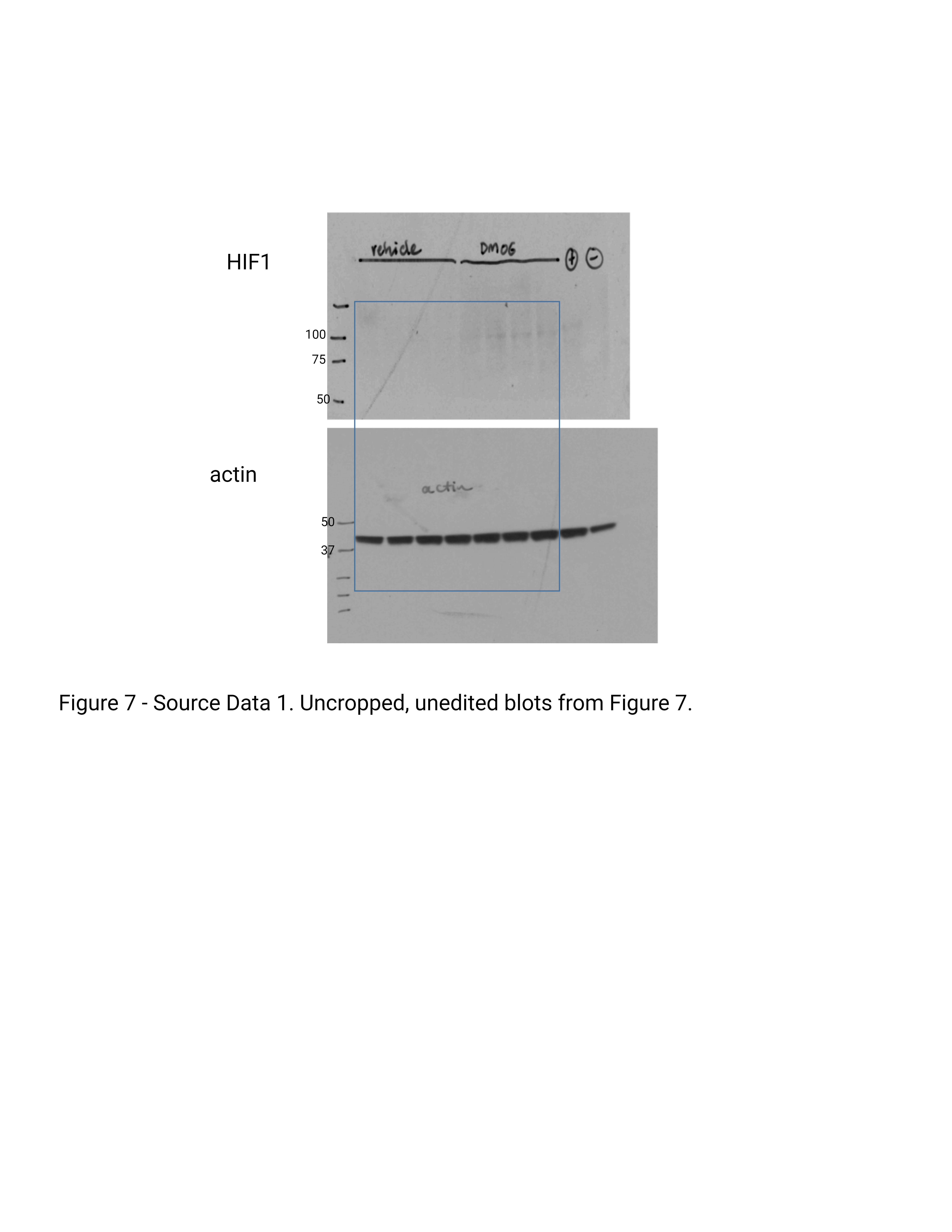

Supplement: Figure 7—source data 1. [file elife-85597-fig7-data1.zip › 85597Figure7-SourceData1.png]
